# A mixed-methods approach to conceptualizing friendships in anorexia nervosa

**DOI:** 10.1371/journal.pone.0254110

**Published:** 2021-09-15

**Authors:** Nandini Datta, Molly Foukal, Savannah Erwin, Hannah Hopkins, Kate Tchanturia, Nancy Zucker

**Affiliations:** 1 Department of Psychiatry and Behavioral Sciences, Stanford University School of Medicine, Stanford, CA, United States of America; 2 Duke University Department of Psychology and Neuroscience, Durham, NC, United States of America; 3 Department of Psychological Medicine, Section of Eating Disorders, Institute of Psychiatry, Psychology and Neuroscience, King’s College London, London, United Kingdom; 4 South London and Maudsley NHS Trust, National Eating Disorders Service, Psychological Medicine Clinical Academic Group, London, United Kingdom; 5 Department of Psychology, Ilia State University, Tbilisi, Georgia; 6 Duke University School of Medicine Department of Psychiatry & Behavioral Sciences, Durham, NC, United States of America; National Institutes of Health, UNITED STATES

## Abstract

**Background:**

Individuals with anorexia nervosa have reported feelings of loneliness, social anhedonia, and interpersonal difficulties. This study sought to clarify the nature of interpersonal relationships in adults with anorexia, which may help improve existing interventions while also facilitating the attainment of something that might compete with the drive for thinness: friendships.

**Methods:**

The present study used a mixed-methods approach to investigate friendship experiences in three groups: anorexia (n = 27), participants with a history of anorexia who are weight restored (n = 20), and healthy controls (n = 24). Thematic analysis was used to isolate the most prevalent themes that emerged from an open-ended interview of experiencing friendships in a subset of participants. Three self-report questionnaires investigating friendship valuation and attachment styles were also administered.

**Results:**

11 unique themes emerged in the data: social comparison, reciprocity, trust, fear of negative evaluation, perceived skills deficit, logistical barriers, reliability, identity issue, low interest, similarity, and conflict avoidance. Only 17% of those with anorexia reported experiencing friendships as positive, relative to 82% of healthy controls and 52% of weight restored participants. Lastly, on self-report measures, participants with anorexia reported greater reliance on themselves versus others, greater use of care-seeking behaviors, and more fear/anger at the thought of losing an attachment figure (p < .05 in all cases).

**Conclusion:**

Results suggest that individuals with anorexia have particular challenges which interfere with the formation and maintenance of friendships, such as viewing friendships negatively and struggling with social comparisons in friendships. Assessing and addressing barriers to intimacy may motivate those with anorexia to relinquish dangerous symptoms that maintain the illness.

## Introduction

Anorexia nervosa (AN) is an isolating psychiatric disorder. It is characterized by severe weight loss resulting from behaviors such as purging, excessive exercise, and dietary restraint, and is accompanied by disturbances in the manner by which the body is experienced [1, 2]. Interpersonal features increasingly reported in those with AN include difficulty discerning the perspectives of others, isolation from previously established relationships, and loneliness [3, 4]. While these features are not directly related to shape and weight concerns, they may complicate the course of the disorder. Results from treatment trials are increasingly encouraging in demonstrating improvement of clinical outcomes both at end-of-treatment and through follow-up periods [5, 6]. Yet, there remains a significant subset of individuals who fail to respond to the most promising interventions. Once the duration of AN exceeds three years, the proportion of individuals who respond to treatment significantly diminishes [7]. To date, effective treatments in AN are relational, requiring communication with a therapist and often incorporating a family component. Clarifying the nature of interpersonal functioning seen in adults with AN may not only help to improve existing interventions but may also help facilitate the attainment of something that might compete with the drive for thinness in AN: friendship [2].

Friendships are an index of healthy development [8, 9]. In a non-clinical sample, Mullinax and colleagues (2016) found that greater satisfaction derived from close friendships was associated with increases in healthy lifestyle choices, such as reciprocal encouragement of regular eating patterns and exercise. In individuals with AN, friendships have been shown to increase motivation for recovery [10, 11]. Malmendier-Muehlschlegel, Rosewall [11] found that in an inpatient facility for adolescents with AN, intimacy, encouraging others, and receiving help was associated with readiness to change. Additionally, the process of recovery from AN has been found to be associated with a reclamation of a previously fragmented sense of self, in part, through the establishment of meaningful relationships [10]. While recovery from AN is understandably complex, it appears that positive friendships may assist in promoting recovery, perhaps by providing individuals with a stronger sense of self.

Despite these reported benefits of friendships, qualitative and mixed methods data have found evidence of social challenges in this population preceding the onset of the disorder. For example, 70% of a sample of 90 adults with AN reported that early social difficulties pre-dated the onset of their illness [12]. Having smaller social networks, feeling disconnected from peers, and reporting difficulties understanding friendships have been cited as challenges occurring prior to the eating disorder (ED) onset [13, 14]. These data are aligned with the cognitive-interpersonal maintenance model of AN, which suggests that social-emotional deficits both predispose AN and are exacerbated during the ill state [15, 16].

These difficulties have been found to worsen during the ill state [17, 18]. Possible behavioral patterns that may contribute to these decrements include social isolation and competition–two patterns that may mutually reinforce each other. For example, individuals with AN report frequent engagement in comparisons to someone perceived as “better” [19, 20]. Such comparisons have been found to contribute to lower self-esteem and internalization of the thin ideal [19], potentially reinforcing social isolation. In a pilot study of adults with severe and enduring AN (>10-year illness history), interpersonal avoidance was found to result in significant impairment of social functioning [21]. These difficulties may contribute to symptom maintenance in AN, and when left untreated, result in an impaired quality of life in the domain of social relationships, comparable to other severe and enduring mental illnesses [21]. The aspects of friendships those with AN find challenging that may motivate social avoidance is little understood, such data is needed to inform interventions targeting interpersonal functioning.

Indeed, characterizing challenges that hinder successful interpersonal functioning amongst individuals with AN is critical for several reasons. First, therapy is an inherently social process, and greater knowledge about the nature of interpersonal relationships in AN may help improve existing treatments by informing the patient-therapist dynamic. Second, it is important to understand whether interpersonal success can be used as a motivational factor for recovery. One seemingly insurmountable obstacle for effective treatment of AN is the identification of sources of reinforcement that prove more valuable than the drive for thinness. Recovery from AN often necessitates the individual expand their life to include other valued activities, which can be difficult due to how highly the illness itself is valued [22, 23]. Supportive relationships have been highlighted as an important factor in recovery [23]. However, it is unclear whether individuals with AN value friendships and relatedly, whether friendships would be sufficient competition for the disorder during recovery. Existing literature investigating social functioning in AN have been done in adolescent samples or small, underpowered samples of adults, citing difficulties with generalizability as a limitation [13]. It is important to assess friendships in adequately-powered adult samples since the severity and endurance of AN in adulthood often leads to treatment resistance [11, 24, 25]. No literature to our knowledge has attempted to identify specific challenges people with AN describe as associated with friendships; such data is an important preliminary step in better understanding the characterization of friendships in AN.

Thus, the present study utilizes a mixed-methods approach to investigate differences in friendship characteristics and valuation between three groups: a clinical group (AN), a weight-restored group with a history of AN (AN-WR), and a healthy control group (HC). A weight-restored group was included to investigate the impact of friendships at different stages of illness. Due to the potential confounding impact of weight on analyses, we felt it necessary to include a weight-restored group in our analyses for a more comprehensive investigation. This study seeks to clarify whether people with AN describe friendships more negatively or positively than AN-WR and HC participants, and what challenges in particular may be cited as contributing to their experience through answering open-ended questions regarding friendship experience and completing self-report surveys. Informed by existing data, we hypothesize that the AN group will report more negatively valanced responses when describing what it is like being in a friendship relative to the AN-WR and HC groups. Relatedly, we hypothesize that individuals with AN will nominate social comparisons as a barrier to maintaining and forming relationships [19, 26]. We anticipate that these qualitative data will be supported by differences in survey responses probing the enjoyment of close friendships, the need for social relationships, and patterns of attachment. Based on existing literature, we anticipate individuals with AN may cite less enjoyment derived from friendships and a greater reliance on oneself versus others [27].

## Materials and methods

### Participants

Participants were recruited using flyers advertising a study on relationships distributed on campus at a Southeastern private university and nearby state universities, eating disorder (ED) focused websites, and by local healthcare providers specializing in EDs. For the current study, we will only report results from a subset of the entire battery administered. Further details about the testing protocol are published elsewhere [28]. The Duke University Institutional Review Board approved this study and written consent was obtained from all adult participants.

Individuals were assigned to one of three groups, including those with a current diagnosis of AN, AN-WR with a history of AN, and HCs; (n_AN_ = 27, n_AN-WR_ = 20, n_HC_ = 24). A subset of these participants completed open-ended interviews regarding their experience in friendships; (n_AN_ = 12, n_AN-WR_ = 15, n_HC_ = 22). Participants were included in the current AN category if they met DSM-IV diagnostic criteria at the time of the interview and in the AN-WR category if they had previously met AN criteria but were fully weight restored for at least six months. Males were excluded from the larger study [28] in which biological sex was a confounding variable. Further, individuals exhibiting current symptoms of psychosis, a thought disorder, substance abuse, or a learning disability were also excluded. Healthy controls had no current or prior history of ED symptomology. Clinical and demographic characteristics can be found in Tables 1 and 2 for the entire sample, (please reference S1 and S2 Tables for information specific to the subgroup (*n* = 49) that participated in the qualitative interview).

**Table 1:** Sample descriptive information (n = 71).

Variables: Mean (SD)	Current AN (n = 27)	WR (n = 20)	HC (n = 24)
Age (years)	27.63 (8.52)	27.35 (10.15)	26.25 (9.74)
BMI (kg/m^2^)	17.35 (1.27)	21.44 (2.12)	22.60 (3.77)
Years of Education^†^	15.12 (2.43)	16.32 (3.33)	15.63 (3.19)
High School or Less	4 (14.8%)	3 (14.3%)	3 (12.5%)
College	14 (51.95)	7 (33.3%)	12 (50%)
Graduate	6 (22.2%)	9 (42.9%)	9 (37.5%)
Race			
White	24 (88.9%)	18 (85.7%)	14 (58.3%)
Black	0	1 (4.8%)	5 (20.8%)
Asian	0	1 (4.8%)	1 (4.2%)
Hispanic	0	0	1 (4.2%)
Mixed	0	0	1 (4.2%)
Other	0	0	2 (8.3%)
Relationship Status			
Single, partnered	4 (14.8%)	5 (23.8%)	11 (45.8%)
Single, not partnered	14 (51.9%)	9 (42.9%)	9 (37.5%)
Separated	1 (3.7%)	0	0
Engaged	0	1 (4.8%)	0
Divorced	1 (3.7%)	0	0
Married	3 (11.1%)	5 (23.8%)	4 (16.7%)

^†^Years of education includes completed and currently in progress.

**Table 2:** Sample clinical characteristics (n = 71).

Variables: Mean (SD)	Current AN (*n = 27*)	WR (*n = 20*)	HC (n = 24)
EDE-Q Global Score	4.12 (1.00)	2.14 (1.04)	.87 (.70)
Anxiety (State)*	51.21 (12.18)	35.67 (7.93)	30.10 (7.89)
Anxiety (Trait)*	58.50 (9.11)	42.38 (10.22)	33.17 (8.86)
Age of Onset (years)	17.48 (4.33)	15.32 (4.12)	-
Subtype			
Binge	1 (3.7%)	2 (10%)	0
Purge^†^	3 (11.1%)	0	0
Binge/Purge	1 (3.7%)	0	0
Restrictive	22 (81.48%)	0	0
Lowest BMI (SD)	14.94 (1.52)	15.18 (1.65)	20.40 (2.12)
Duration	*n* = 22	*n* = 13	
Years at low weight	9.77 (9.04)	5.62 (7.59)^‡^	-
# Years weight restored	-	9.23 (6.89)	-

*There are significant differences (p ≤ .05) in continuous measures of state and trait anxiety between groups, consistent with reports of long-term outcomes of AN in the current literature [29].

^†^Including engagement in the following compensatory behaviors: misuse of laxatives, diuretics, excessive exercise, and self-induced vomiting episodes.

^‡^One AN-WR participant reported having been at their lowest weight for 29 years, which falls over three standard deviations above the mean; without this data point, the mean duration of low weight for AN-WR is 3.58 (3.12).

### Testing procedures

All participants completed a standardized two-day assessment, a subgroup of these participants also completed an open-ended interview of relationship functioning (n = 49) [28]. The open-ended survey regarding experiences in friendships included the following questions: “What is the most challenging thing for you about being in a friendship?”; “Are there particular challenges with same age, same gender friendships?”; and “What it is like for you being in a friendship?” The interviewer transcribed participant responses using word processing software. Once data were de-identified, all responses were uploaded and stored securely using the qualitative research software platform *NVivo®*.

### Qualitative data analysis

Researchers employed thematic analysis to examine participants’ responses to the three interview items described above [30]. Two independent coders reviewed all qualitative data closely and identified overarching themes that emerged across participants’ responses. In an initial stage of inductive coding, researchers worked separately to uncover initial concepts present in the raw data. Once open coding reached a saturation point (i.e., no new codes could be generated), researchers went back through each response to ensure all codes were applied uniformly. At this point, researchers compared codebooks and emergent themes. The researchers collaboratively identified and resolved discrepancies in coding and established a new unified coding system. This was used to re-code all interview responses. Next, researchers met to compare results, during which all discrepancies were reconciled. Lastly, interrater reliability was calculated with Cohen’s kappa statistics and determined to be 87.5%, which is considered to be good agreement [31].

### Quantitative data analysis

Analyses of variance were used to probe differences between groups on the self-report measures given. The current study was sufficiently powered to detect medium-large effects between groups, (1 - β = .96). For all analyses, *p*-values are reported, but due to the exploratory nature of the study, we also provide effect sizes for interpretation [32]. All post-hoc analyses used Bonferroni post-hoc criterion to probe pairwise comparisons between groups.

### Measures

#### Cambridge Friendship Questionnaire–CFQ [33]

The CFQ is used to assess the motivation for and enjoyment of close friendships for adults. The CFQ is scored from 0–135, higher scores reflect greater enjoyment, importance, and motivation to pursue friendships. The CFQ is comprised of 35 questions total, 27 of which are scored. Other studies have demonstrated acceptable test-retest reliability (Kappa = .80) and good external validity [34]. In our sample, internal consistency for the CFQ was moderate (α = .75).

#### Affective Relationship Scale–ARS [35]

The ARS characterizes an individual’s social network and the relative health of familial relationships, intimate partnerships, and friendships. The ARS is designed to yield two [33] scores: one total score for each figure (reflecting the strength of one’s need for relationships) and one set of sub-scores assessing seeking proximity; receiving emotional support; receiving reassurance for behavior; receiving encouragement and help; sharing information and experience; and giving nurturance. These scores reflect the impact that figure has on one’s life. For our sample, internal consistency was good (α = .89).

#### Reciprocal Attachment Questionnaire–RAQ [36]

The RAQ is a 43-item self-report measure that assesses a person’s pattern of attachment to an individual with whom a relationship has been shared for at least a period of six months. The RAQ provides five subscales and four pattern scores, capturing a representation of the individual’s attachment system and the degree of pathology present in an individual’s attachment pattern. The five subscales include perceived availability, proximity seeking, separation protest, feared loss, and use of attachment figure. The four pattern scales include compulsive self-reliance, compulsive care-giving, compulsive care-seeking, and angry withdrawal. In our sample, internal consistency for the RAQ across groups was moderate (α = .71).

#### State Trait Anxiety Inventory–STAI [37]

The STAI is a 20-item self-report questionnaire assessing state and trait anxiety, where greater scores reflect greater levels of anxiety endorsed. In the present sample, internal consistency was fair (α = .65). Norms from a sample of 481 female adults, were (*M* = 38.76, *SD* = 11.95, α = .93) for state, and (*M* = 40.40, *SD* = 10.15, α = .91) for trait anxiety [37]. Good psychometric properties of the STAI have been shown, with internal consistency coefficients for the scale ranging from .86 to .95 [37]. In our sample, internal consistency for the STAI across groups was (α = .60).

#### Eating Disorder Examination-Questionnaire–EDE-Q [38]

The global EDE-Q score is comprised of 23 questions assessing the frequency of ED behavior over the past 28 days. These items are rated on a 7-point forced choice scale, from 1 (“No days”) to 7 (“Every day”). The global pathology score is comprised of the average of four subscales: Eating restraint (5 questions), eating concern (5 questions), shape concern (8 questions), and weight concern (5 questions) subscales. Global and subscale scores greater than *(M* = 4.02, *SD* = .28) are considered to be clinically significant and indicates a person’s chronic engagement in ED behaviors, thoughts, or feelings [39]. In our sample, internal consistency for the global score was (α = .83).

## Results

### Qualitative results

#### General friendship challenges with same-aged/gender peers

**Fig 1:**
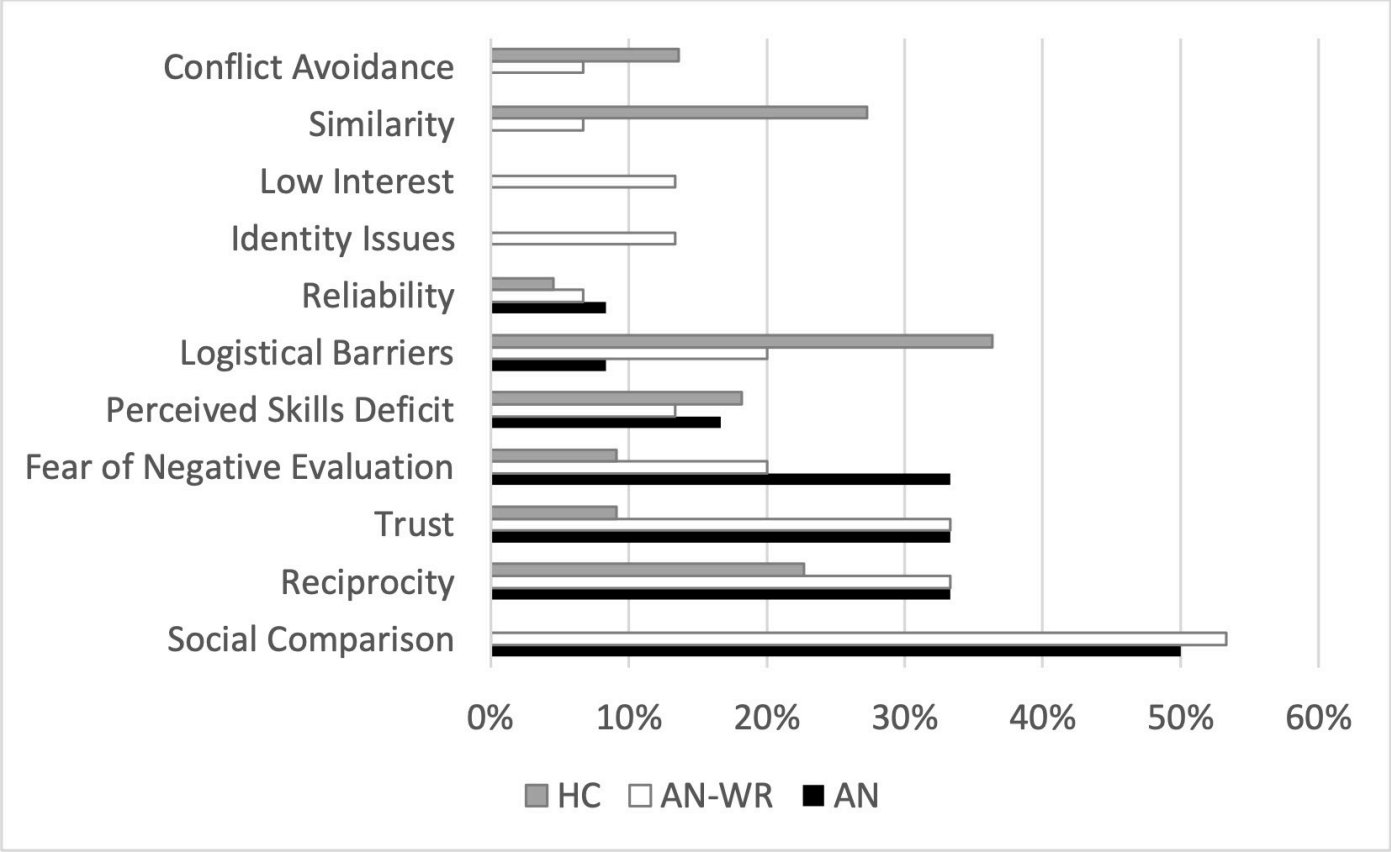
Frequency of themes: Greatest challenges in friendships. This figure displays the frequency of themes that emerged to a question querying about general challenges with friendships by group (HC, AN-WR, and AN). The y-axis is each of the 11 themes that emerged (out of 11 total), and the x-axis is percent of each group which endorsed the theme. In this figure, the black bar represents those in the AN group, the white bar represents those in the AN-WR group, and the grey bar represents those in the HC group.

Eleven unique themes emerged from the data during the process of qualitative analysis in response to the question probing challenges with friendships (Table 3). Fig 1 displays the frequency of these themes by group in response to a question querying about general challenges with friendships. Social Comparison emerged as one of the most frequently applied themes; however, it was only observed among those in AN or AN-WR groups, specifically within the context of particular challenges with same age, same gender friendships. Although several HC participants did acknowledge competition as an issue, none of these individuals described themselves as competitive; instead, they noted how others had exhibited this behavior. For example, one HC participant noted, “I always think it is their problem and not mine. I sense that they have a problem with me and now that I am older, I try to get out of their way and stay low.” Another HC echoed similar sentiments, “I have had instances where I felt my friends competed with me unnecessarily and we ended up not being friends.”

**Table 3:** Qualitative codes and example text.

Code	Example
**Social Comparison**	*“Usually have a tendency to compare*.*”*
	*“Feel insecure… when I have friendships in which person is good at what I am good at*. *Used to be hard in relationships with thin people because always comparing*.*”*
	*“Somehow I feel like they are more mature or older than me*. *I feel like a child at times compared to them*.*”*
	*“Maybe I just feel more judged by girls*. *Too much competition*. *That competition sort of makes my old thoughts become an issue*.*”*
**Reciprocity**	*“I always feel like I am giving*, *giving*, *giving and I don’t feel like I am getting anything back*.*”*
	*“Being a giver and not just a taker… I just feel that I am always on the receiving end…Like what can you do for me*.*”*
**Trust**	*“In the beginning*, *may take longer to generate trust and thus am very reserved*.*”*
	*“Trust*. *I always find it hard to trust people*. *I always think they are lying to me*. *Always*.*”*
**Fear of Negative Evaluation**	*“Would fear rejection if I were honest*.*”*
	*“Thinking others will be judgmental or won’t want to hear about it… that others don’t want to be bothered*.*”*
	*“I can’t express how I feel sometimes for fear of driving them away*.*”*
**Perceived Skills Deficit**	*“Sometimes I feel I can’t communicate effectively what I want to say*.*”*
	*“I get really awkward in social situations–more than most people*.*”*
**Logistical Barriers**	*“Making time to get together*. *Other stuff to do gets in the way*.*”*
	*“Keeping in contact if not living in the same city*.*”*
**Reliability**	*“Keeping commitments*. *Not canceling*.*”*
	*“I find it hard to follow through with commitments as I don’t manage my time very well*.*”*
**Identity Issues**	*“Figuring out what they want and molding myself into that*.*”*
	*“Preserving emotional boundaries and not becoming emotionally enmeshed… see self as passive and others as more seeking*.*”*
**Low Interest**	*“Maybe I don’t initiate enough contact*. *Tend to be more passive… Usually just doesn’t occur to me*.*”*
**Similarity**	*“I don’t find a lot of friendships with women of my age and gender because I don’t have a lot in common*.*”*
	*“We have different life priorities*.*”*
**Conflict Avoidance**	*“I might tend to avoid the conflict without talking about it*.*”*
	*“Afraid that if I am totally honest that sometimes I will hurt their feelings*.*”*

The most frequently applied code among HC was logistical barriers, such as distance and time (Fig 1). This code was applied half as many times amongst the AN and AN-WR groups combined compared to the HC group. Another challenge cited frequently among HC participants and less frequently among the AN and AN-WR was similarity. Most often this theme emerged among HC participants as a particular challenge with same age, same gender friendships. One HC group member described this, “I don’t find a lot of friendships with women my age and gender because [we] don’t have a lot in common. That’s probably the biggest challenge.” It is possible that individuals without a current or prior AN diagnosis may be more likely to identify external features of friendships such as finding commonalities with the other person and logistics as primary barriers to connecting with a potential friend. For individuals with current or past AN, internal sources of conflict such as social comparison and competition appear to be cited more frequently as the greatest challenge.

The code of reciprocity was coded relatively evenly across groups, suggesting that the challenge of balancing the give and take in a friendship is a common experience regardless of AN diagnosis and symptomology. Also consistent across groups was the code of reliability; however, this code was observed only once for each group. Thus, being reliable and showing up for a friend was a challenge not commonly reported by those with or without AN.

#### Specific friendship challenges with same-aged/gender peers

**Fig 2:**
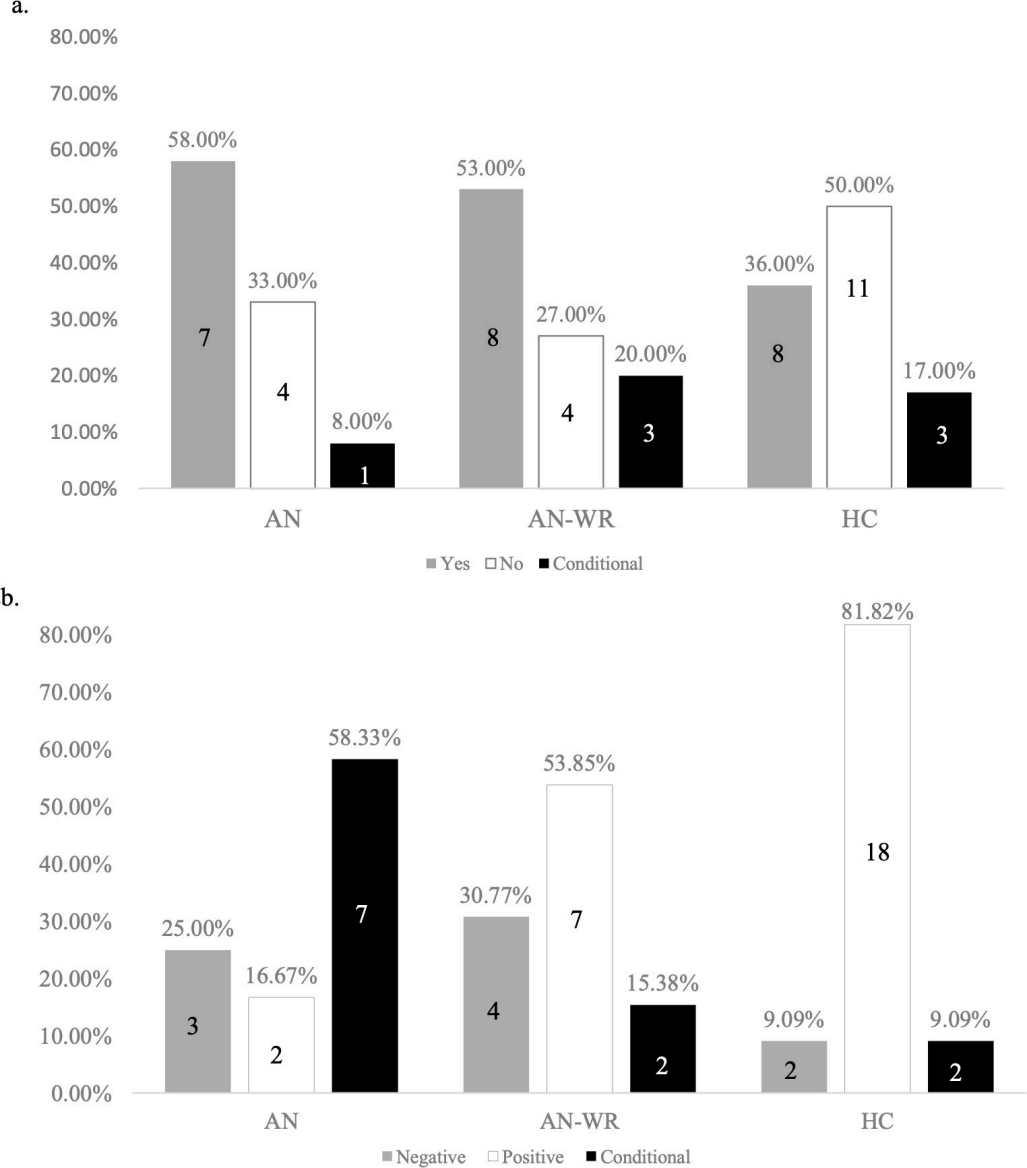
a. Friendship challenges yes/no. Fig 2A depicts how many of each group responded yes/no to Question 2: “Are there particular challenges with same age, same gender friendships?” Here, the y-axis is the percent of each group that answered yes (grey bar), no (white bar), or provided a conditional response (black bar). The specific percentages are outlined on each bar. The x-axis is the group membership. b. Friendship experience valence. Fig 2B depicts the valence of responses to Question 3: “What is it like for you being in a friendship?” Here, the y-axis is the percent of each group that answered negatively (grey bar), positively (white bar), or provided a conditional response (black bar). The x-axis is the group membership.

Participants’ response to, “Are there particular challenges with same age, same gender friendships?” are shown in Fig 2A, arranged by group. Fifty percent of healthy controls reported no particular challenges with same age, same gender friendships, while over 50% of individuals in both the AN (58.3%) and AN-WR (53%) groups responded “yes” to this interview question. Among the AN groups, several individuals mentioned a preference for older individuals, noting, “I don’t really like females of my own age. I like older people” and “I don’t get along with people my age. I am always friends with older people.” Individuals in the AN-WR group offered similar sentiments, “I’m comfortable with older people. Earlier I felt alienated from peers” and “[I] always felt people my own age were less mature and I didn’t fit in.” While two members of the HC group did report friendships with older people, only one of these explicitly described difficulty with same age peers, which was explained as being due to “different life priorities.”

The half of the HC group who endorsed challenges with same age, same gender friends described these challenges neutrally and from a self-referenced standpoint. For instance, one HC participant stated, “I can chat with people easily but don’t think I form close friends easily” and “I guess I don’t have as many same age, same gender [friends] as I should or others do” as well as “I find that my same gender friendships seem to come and go more quickly than opposite gender friendships.” Overall, a greater percentage of individuals with current or history of AN report particular challenges with forming and maintaining friendships in their peer group.

#### Valence of friendship experiences

Valences of responses to question 3, “What is it like for you being in a friendship” is presented by group in Fig 2B. Most healthy controls (82%) responded positively when describing their experience being in a friendship, while few individuals in the AN group (17.3%) answered this way. In fact, members of the AN group were more likely to offer a conditional reply (58.3%) or a negative one (25.3%) than positive. In many of these conditional cases, AN group members described specific requirements in order for friendships to exist and/or be positive; “I don’t really engage in friendships unless I can be completely comfortable” and “It needs to be reciprocal” and “As long as there is trust it is good.” Among individuals in the AN-WR group, over half (54%) gave a positive response, such as “Gives me a sense of purpose and belonging” and “I can be myself” and “Feel like I belong.” At the same time, almost a third of the AN-WR group reported a negative experience of being in a friendship. These individuals described feeling “incompetent,” “superficial,” and unable to be authentic; “I don’t feel comfortable sharing who I am” and “don’t feel that I am being who I am.”

Among HC group members reporting positive experiences, many highlighted the process of sharing, both as “sharing a bond with someone” as well as the act of being open with another person. They noted, “it’s nice to have someone to share your feelings with” and described friendship as an opportunity to “share things and give support and advice” and “share experiences and thoughts and problems.” Other positive responses of HC group members described the experience of feeling supported and having someone “to go to if something is wrong;” “talk to about problems;” “depend on to be there for you;” and “turn to in all situations.” One HC described a scenario, “when I mentioned I was having some difficulties and said I didn’t want help, [my friend] said, why that is what friends are for.” Thus, HC group members who experienced friendship as positive also described a willingness to be vulnerable and open with others.

### Quantitative results

#### Strength of social networks

An analysis of variance showed that there were significant differences between groups for the ARS Friend Subscale (Giving Nurturance), *F*(2, 69) = 3.81, *p* = .02 with a medium-large effect size of Cohen’s *f* = .32. Higher scores on this subscale reflect greater nurturance given to friends (e.g., encouraging friend during times of difficulties and liking friends’ requests for help when in difficulty); Bonferroni post hoc analyses indicate that the AN group had significantly higher scores (M = 9.92, SD = .30) relative to AN-WR participants (M = 9.47. SD = .81), S3 Table. The pairwise comparison of the healthy control scores with the weight restored group and currently AN group were both non-significant.

Additionally, there were significant differences between groups for three RAQ pattern subscales (Compulsive Self-Reliance, Compulsive Care Seeking, and Angry Withdrawal) and two RAQ subscales (Use of Attachment Figure, Feared Loss). All post-hoc analyses used Bonferroni post-hoc criterion to probe pairwise comparisons between groups. These differences are outlined below and in Table 4, while post-hoc analyses and mean differences can be found in S3 Table. Given the exploratory nature of this study, we report effect sizes in addition to traditional p-values [32].

**Table 4:** ANOVA results for ARS-friends subscale and the total CFQ for entire sample.

	Groups Means (SD)					
	AN (n = 25)	WR (n = 21)	HC (n = 24)	*F*	*p*	*Cohen’s f*
*ARS Proximity*	7.84 (2.17)	7.05 (1.91)	7.63 (1.86)	.94	.39	.23
	AN (n = 26)	WR (n = 21)	HC (n = 24)			
*ARS Encourage*	9.54 (.95)	9.19 (.98)	9.21 (.98)	1.01	.37	.16
	AN (n = 26)	WR (n = 21)	HC (n = 24)			
*ARS Experiences*	9.57 (.81)	9.14 (.96)	9.46 (.78)	1.59	.21	.19
	AN (n = 26)	WR (n = 21)	HC (n = 24)			
*ARS Nurture*	9.92 (.39)	9.47 (.81)	9.83 (.48)	3.81	.027*	.32
	AN (n = 26)	WR (n = 21)	HC (n = 24)			
*ARS Reassurance*	9.19 (1.02)	8.62 (1.50)	8.79 (1.21)	1.34	.27	.22
	AN (n = 26)	WR (n = 21)	HC (n = 24)			
*ARS Support*	8.89 (1.92)	8.57 (1.30)	8.75 (1.39)	.20	.81	.10
	AN (n = 26)	WR (n = 19)	HC (n = 24)			
*ARS Need (total)*	55.04 (5.77)	51.73(5.98)	53.67(4.67)	1.97	.15	.56^**++**^
	AN (n = 27)	WR (n = 21)	HC (n = 24)			
*CFQ Total*	83 (19.97)	86.24(13.8)	86.87(21.20)	.31	.73	.31
	AN (n = 24)	WR (n = 21)	HC (n = 24)			
*RAQ Compulsive Self-Reliance*	20.25 (4.10)	16.67 (5)	14.45 (3.83)	11.10	**.00** *	**1.17** ^**++**^
*RAQ Compulsive Care-Giving*	24 (4.32)	22.38 (4.79)	22.13 (4.40)	1.20	.31	.39
*RAQ Compulsive Care Seeking*	19.45 (6.10)	15.38(5.74)	13.17 (3.57)	8.90	**.00** *	**1.17** ^**++**^
*RAQ Angry Withdrawal*	16.79 (4.45)	13.14 (4.41)	14.04 (4.96)	3.88	**.03** *	.72^**++**^
*RAQ Separation Protest*	6.79 (3.05)	6.09 (2.46)	5.79 (1.61)	1.05	.38	.29
*RAQ Attachment Figure Use*	6.87 (2.83)	5.48 (2.10)	4.83 (1.69)	5.13	**.01** *	.58^**++**^
*RAQ Availability & Responsiveness*	7.25 (3.15)	5.85 (1.59)	5.63 (2.14)	3.14	.05	.49^**++**^
*RAQ Proximity Seeking*	8.08 (3.50)	7.62 (3.58)	6.08 (1.76)	2.83	.07	.51^**++**^
*RAQ Feared Loss*	9.21 (3.57)	6.00 (3.38)	5.54 (2.20)	9.86	**.00** *	**.94** ^**++**^

* indicating significance of p < .05 between groups.

^**++**^ large effect sizes: Cohen’s f > 0.4.

Significant differences emerged between the AN group relative to the AN-WR group and HC group for the following RAQ Subscales: Compulsive Self-Reliance, (*F*(2, 69) = 11.10, *p* = .00; Cohen’s *f* = 1.17), indicating that the AN group on average reported greater self-sufficiency relative to reliance on others; Compulsive Care Seeking (*F*(2, 69) = 8.90 *p* = .00; Cohen’s *f* = 1.17), indicating the AN group reported more frequent care-seeking behaviors; and Feared Loss (*F*(2, 69) = 9.86, *p* = .00; Cohen’s *f* = .94.), indicating the AN group reported greater fear of losing an attachment figure. The AN group reported significantly greater Angry Withdrawal scores relative to the AN-WR group (*F*(2, 69) = 3.88 *p* = .03, Cohen’s *f* = .72), where greater scores reflect more negative reactions/anger to perceived loss of an attachment figure. Lastly, the AN group reported greater Use of an Attachment Figure scores relative to the HC group (*F*(2, 69) = 5.13, *p* = .01, Cohen’s *f* = .58), where greater scores reflect greater use of their attachment figure.

No significant differences between groups were found for the Cambridge Friendship Questionnaire (*p* = .73).

## Discussion

This mixed-methods study of friendships in individuals with AN revealed three important findings that will each be discussed in turn. First, those with AN report more negative and less positive experiences from friendships and have particular challenges with same age, same gender peers. Second, those with AN, either currently or weight-restored, report themes of social comparison as a challenge to friendship formation and maintenance. Finally, there seems to be internal conflict in those with AN between how they want to behave in a friendship versus how they perceive themselves as behaving, a conflict that may diminish the rewarding value of friendships.

Perhaps most critically, only 17% of individuals diagnosed with AN reported friendships as being positive relative to 82% of HC and 52% of AN-WR. Clarification of barriers to friendships may offer some clues as to why friendships are not perceived more positively in those with AN. The theme endorsed most frequently in those with current AN and AN-WR was that of social competition. The cross-sectional and single informant design of this study cannot provide causal information as to why those with AN experience friendships less positively than healthy controls. However, it does provide evidence to guide future longitudinal investigations. Current findings highlight that those with AN may internalize specific prerequisites or conditions that must be present in order to feel comfortable in a friendship. This ‘conditional’ perspective toward friendship may stem from an expectation that the other person will reject or abandon them, supported both by the significantly higher fear of loss and use of attachment figures reported among the AN group in the quantitative results as well as prior literature [40]. These perspectives and fears may be heightened by elevated state and trait anxiety evidenced in individuals with AN [29, 41]. As a result, this group may engage in more reassurance-seeking behavior to confirm they are important to the other person, such as compulsive care seeking, which was significantly higher among the AN group in both the present study and in prior research [27].

Results of the present study also suggest that individuals with current or prior AN may be more likely to cite social comparison as the greatest challenge in friendship, while those with no history of AN may be more likely to cite logistical barriers. Individuals struggling with AN often engage in harsh self-criticism and view themselves through a lens of constant evaluation surrounding achievement, performance, or productivity [42]. In this mindset, others’ accomplishments may be perceived as a threat, as this population may be more prone to making self-defeating appraisals [43]. Thus, individuals struggling with current or prior AN may view others primarily as a source of competition, and in turn, experience interpersonal connection as a trigger for increased self-criticism. This would also help explain our finding that relative to healthy controls, AN and AN-WR individuals appear to struggle more in same age, same gender friendships, and seek out friendships with older people. Perhaps the rationale of age difference helps create perceived distances that may decrease social comparison, thereby reducing perceived threat and making the experience of friendship less aversive.

Finally, healthy controls reporting positive friendship experiences were more likely to describe a willingness to be vulnerable and open about their own struggles, a trait not echoed among individuals in the AN group. Instead, individuals with AN reported significantly higher scores on the compulsive self-reliance subscale, consistent with prior research [27], suggesting that this group may be more likely to rely only on themselves in times of need or uncertainty. Given the significantly higher fear of loss reported among the AN group, this notion of perceived self-sufficiency may serve an important protective function. In addition, it aligns with the ‘conditional’ nature of friendships reported amongst individuals with AN in the present study; unless trust is absolutely certain, relying on one’s self feels much safer than risking disappointment or loss. Nevertheless, this reluctance to share one’s struggles may create a barrier for building meaningful connection with others. Revealing one’s imperfections may help one appear more relatable and even more likeable; thus, individuals who are unwilling to exhibit any flaws may experience greater difficulty attracting potential friends. Further, being vulnerable with another can create a shared sense of common humanity, reducing feelings of isolation while deepening connection. As a result, the experience of friendship may feel more positive and rewarding, generating a positive feedback loop motivating ongoing vulnerability and interpersonal engagement. On the other hand, a friendship devoid of vulnerability and sharing may lack the reinforcement necessary to truly value or maintain over the long-term.

The current study has several limitations to be considered. The self-report nature of this exploratory study may be vulnerable to response bias. The semi-structured interview was created with the intention of allowing subjects to provide free-form responses without imposing significant restrictions on the topics they addressed and has not been validated by prior research. The sample size for this study is small, though effect sizes and confidence intervals were calculated to further explain the magnitude of findings. Due to the small sample size, generalizability of the findings reported in this manuscript are limited.

The clinical implications of the current findings hold potential value for informing existing treatments for adult AN. These preliminary findings may shed some light on additional treatment targets to support improvement of interpersonal difficulties in individuals with AN. Specifically, clinical interventions may benefit from the incorporation of work reducing social comparisons and increasing self-compassion, to help individuals with AN find value in friendships and social support, the latter of which has been found to be helpful in recovery from an eating disorder [18]. Group therapy focused on an integration of self-compassion, practicing vulnerability, and engaging in a shared sense of common humanity may be particularly beneficial for adults in conjunction with individual psychotherapy. There is limited data on group interventions for adults with AN; the preliminary data presented in the current manuscript holds promise for future efforts to assess the impact of such treatments on recovery in this population.

These findings begin to elucidate what challenges specifically interfere with the formation and maintenance of friendships in those AN, as compared to those with a history of AN who are weight restored and healthy controls. Friendships are viewed more negatively in those with AN, and social comparison was cited as a unique challenge to friendships in those with AN and with a history of AN. These findings, in conjunction with individuals with AN reporting more compulsive self-reliance and less willingness to be open and vulnerable in friendships, begins to explain some reasons why friendships may be perceived as less rewarding in this population.

## Supporting information

S1 Table Qualitative sample descriptive statistics. Descriptive demographics for participants who completed the qualitative interview.
(DOCX)

S2 Table Qualitative sample clinical characteristics. Clinical characteristics for participants who completed the qualitative interview.
(DOCX)

S3 Table Post-hoc comparisons. Post-Hoc ANOVA comparisons of significance.
(DOCX)
