# Supplementary material for: A mixed-methods approach to conceptualizing friendships in anorexia nervosa
Source: PLoS One. 2021 Sep 15;16(9):e0254110. doi: 10.1371/journal.pone.0254110 (PMC8443043; doi:10.1371/journal.pone.0254110)
Supplement: S1 Table — Descriptive demographics for participants who completed the qualitative interview. (DOCX) [file pone.0254110.s001.docx]

S1 Table. Descriptive statistics for qualitative sample (n=48).

| Variables: Mean (SD) | Current AN  (*n=12*) | WR  (*n=15*) | HC  (n=22) |
| --- | --- | --- | --- |
| Age (years) | 28.17 (9.28) | 30.36 (10.74) | 25.45 (8.46) |
| BMI (kg/m^2^) | 17.58 (1.60)* | 21.38 (2.02) | 22.11 (2.83) |
| Years of Education^a^ | 15.67 (2.49) | 17.00 (3.53) | 15.82 (3.26) |
| High School or Less | 1 (8.3%) | 2 (13.3%) | 3 (13.6%) |
| College | 8 (66.7%) | 3 (20.0%) | 10 (45.5%) |
| Graduate | 3 (25%) | 8 (53.3%) | 9 (40.9%) |
| Race |  |  |  |
| White | 12 (100%) | 12 (80.0%) | 12 (54.5%) |
| Black | 0 | 1 (6.7%) | 5 (22.7%) |
| Asian | 0 | 1 (6.7%) | 1 (4.5%) |
| Hispanic | 0 | 0 | 1 (4.5%) |
| Mixed | 0 | 0 | 1 (4.5%) |
| Other | 0 | 0 | 2 (9.1%) |
| Relationship Status |  |  |  |
| Single, partnered | 2 (16.7%) | 3 (20%) | 10 (45.5%) |
| Single, not partnered | 8 (66.7%) | 5 (33.3%) | 9 (40.9%) |
| Separated | 1 (8.3%) | 0 | 0 |
| Engaged | 0 | 1 (6.7%) | 0 |
| Divorced | 0 | 0 | 0 |
| Married | 1 (8.3%) | 5 (33.3%) | 3 (13.6%) |

Notes: ^a^ Years of education includes completed and currently in progress.
