# Supplementary material for: A mixed-methods approach to conceptualizing friendships in anorexia nervosa
Source: PLoS One. 2021 Sep 15;16(9):e0254110. doi: 10.1371/journal.pone.0254110 (PMC8443043; doi:10.1371/journal.pone.0254110)
Supplement: S2 Table — Clinical characteristics for participants who completed the qualitative interview. (DOCX) [file pone.0254110.s002.docx]

S2 Table*.* Clinical characteristics for qualitative sample (n=48).

| Variables: Mean (SD) | Current AN  (*n=12*) | WR  (*n=15*) | HC  (n=22) |
| --- | --- | --- | --- |
| EDE-Q Global Score | 4.19 (.62) | 2.01 (1.10) | .82 (.71) |
| Anxiety (State) | 2.54 (.58) | 1.69 (.39) | 1.49 (.40) |
| Anxiety (Trait) | 2.91 (.36) | 2.11 (.50) | 1.64 (.68) |
| Age of Onset (years)  Subtype | 17.33 (3.10) | 15.86 (5.10) | - |
| Binge | 1 (8.3%) | 0 | 0 |
| Purge^a^ | 1 (8.3%) | 0 | 0 |
| Binge/Purge | 1 (8.3%) | 0 | 0 |
| Restrictive | 9 (75%) | 0 | 0 |

^a^Including engagement in the following compensatory behaviors: misuse of laxatives, diuretics, excessive exercise, and purging episodes.
