# Supplementary material for: A mixed-methods approach to conceptualizing friendships in anorexia nervosa
Source: PLoS One. 2021 Sep 15;16(9):e0254110. doi: 10.1371/journal.pone.0254110 (PMC8443043; doi:10.1371/journal.pone.0254110)
Supplement: S3 Table — Post-Hoc ANOVA comparisons of significance. (DOCX) [file pone.0254110.s003.docx]

S3 Table. Post-Hoc ANOVA Comparisons of Significance

|  | Group | Mean | *SD* | Post hoc Bonferroni | *p* |
| --- | --- | --- | --- | --- | --- |
| ARS Friends Giving Nurturance | AN (1) | 9.92 | .39 | 1>2 | .03 |
|  | WR (2) | 9.47 | .81 | 2<1 | .03 |
|  | HC (3) | 9.83 | .48 |  |  |
| RAQ Compulsive Self-Reliance | AN (1) | 20.25 | 4.10 | 1>2, 1>3 |  |
|  | WR (2) | 16.67 | 5.00 | 2<1 | .02 |
|  | HC (3) | 14.45 | 3.83 | 3<1 | .03 |
| RAQ Compulsive Care Seeking | AN (1) | 19.45 | 6.10 | 1>2, 1>3 |  |
|  | WR (2) | 15.38 | 5.74 | 2<1 | .03 |
|  | HC (3) | 13.17 | 3.57 | 3<1 | .00 |
| RAQ Angry Withdrawal | AN (1) | 16.79 | 4.45 | 1<2 | .03 |
|  | WR (2) | 13.14 | 4.41 | 2>1 | .03 |
|  | HC (3) | 14.04 | 4.96 |  |  |
| RAQ Attachment Figure | AN (1) | 6.87 | 2.83 | 1>3 | .01 |
|  | WR (2) | 5.48 | 2.10 |  |  |
|  | HC (3) | 4.83 | 1.69 | 3<1 | .01 |
| RAQ Feared Loss | AN (1) | 9.21 | 3.57 | 1>2, 1>3 |  |
|  | WR (2) | 6.00 | 3.38 | 2<1 | .01 |
|  | HC (3) | 5.54 | 2.20 | 3<1 | .00 |

*Note.* The numbers in parentheses in group names refer to the numbers used in illustrating statistically significant differences in the column “Post-hoc Bonferroni”
